# Supplementary figures and images for: An Alzheimer‐associated TREM2 variant occurs at the ADAM cleavage site and affects shedding and phagocytic function
Source: EMBO Mol Med. 2017 Aug 30;9(10):1356–65. doi: 10.15252/emmm.201707672 (PMC5623859; doi:10.15252/emmm.201707672)

C

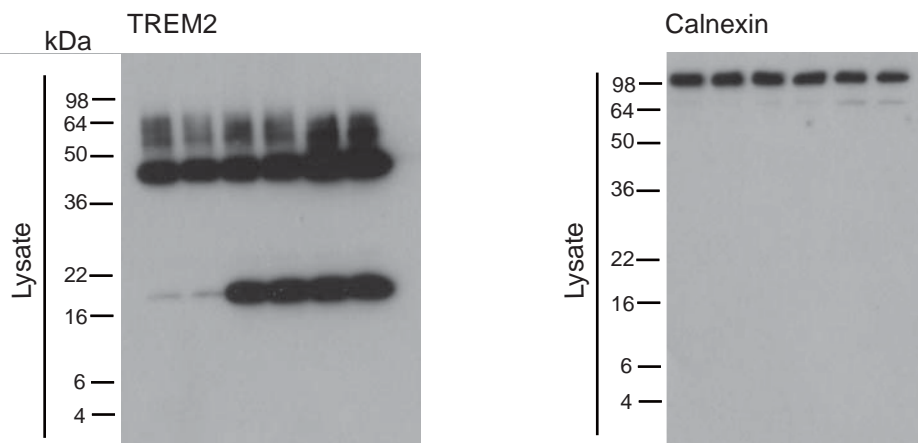

E

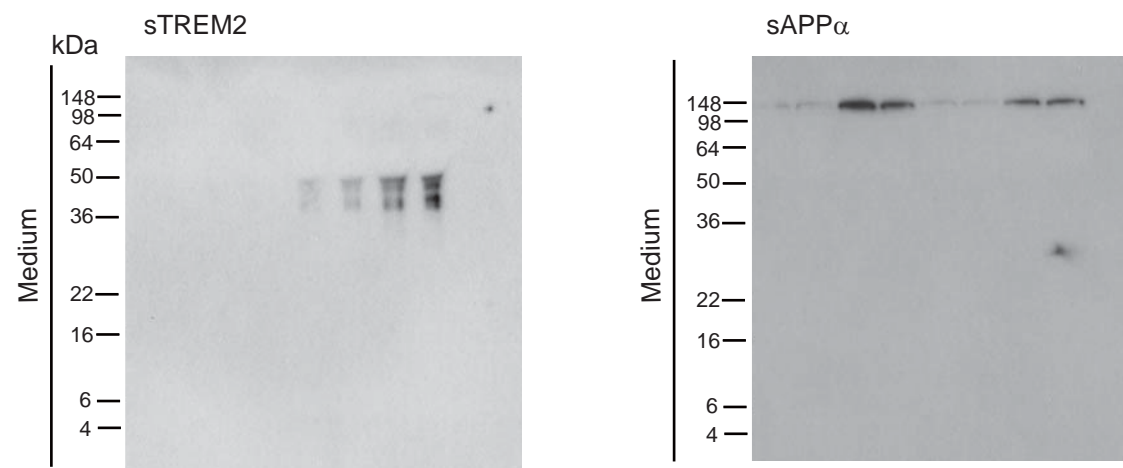

H

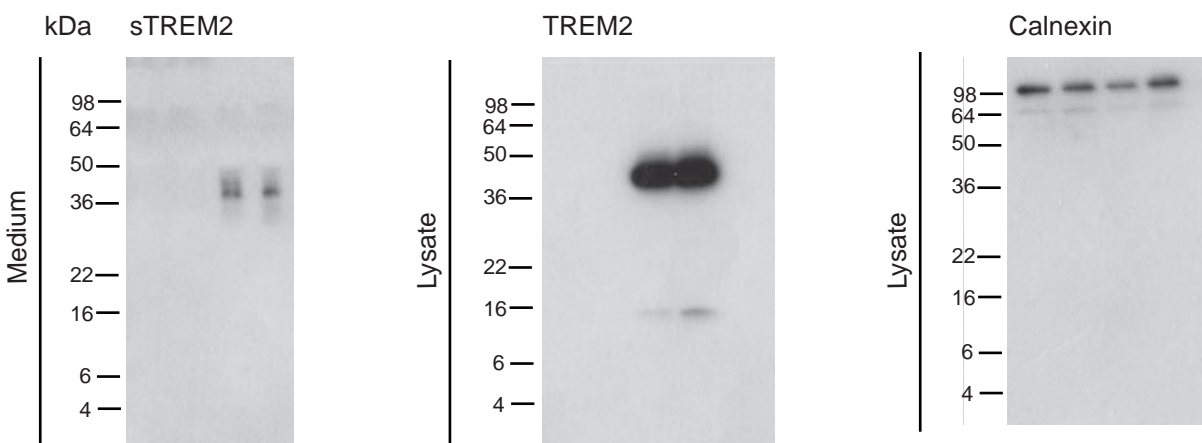

K

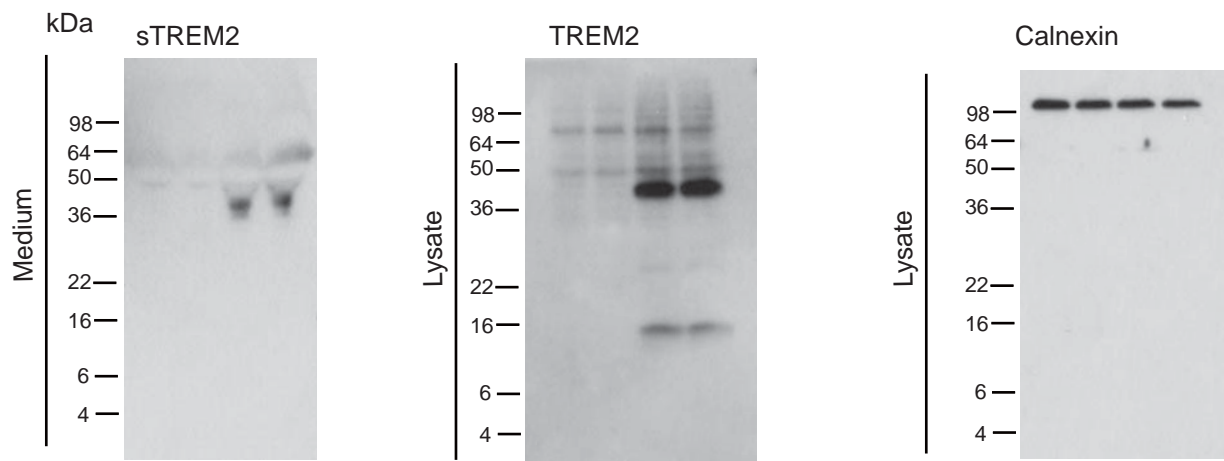

Supplement: Supplementary file 2 — Source Data for Figure 1 [file EMMM-9-1356-s001.pdf]

**B**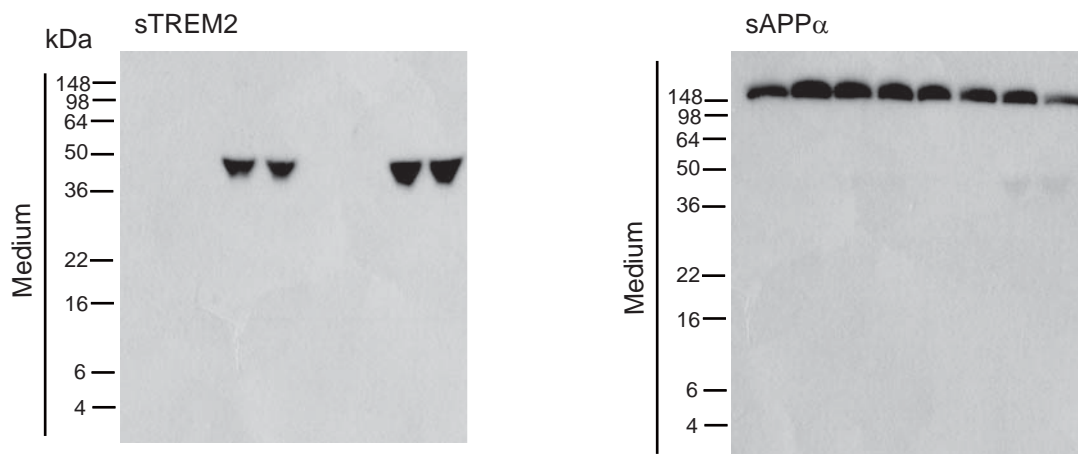**F**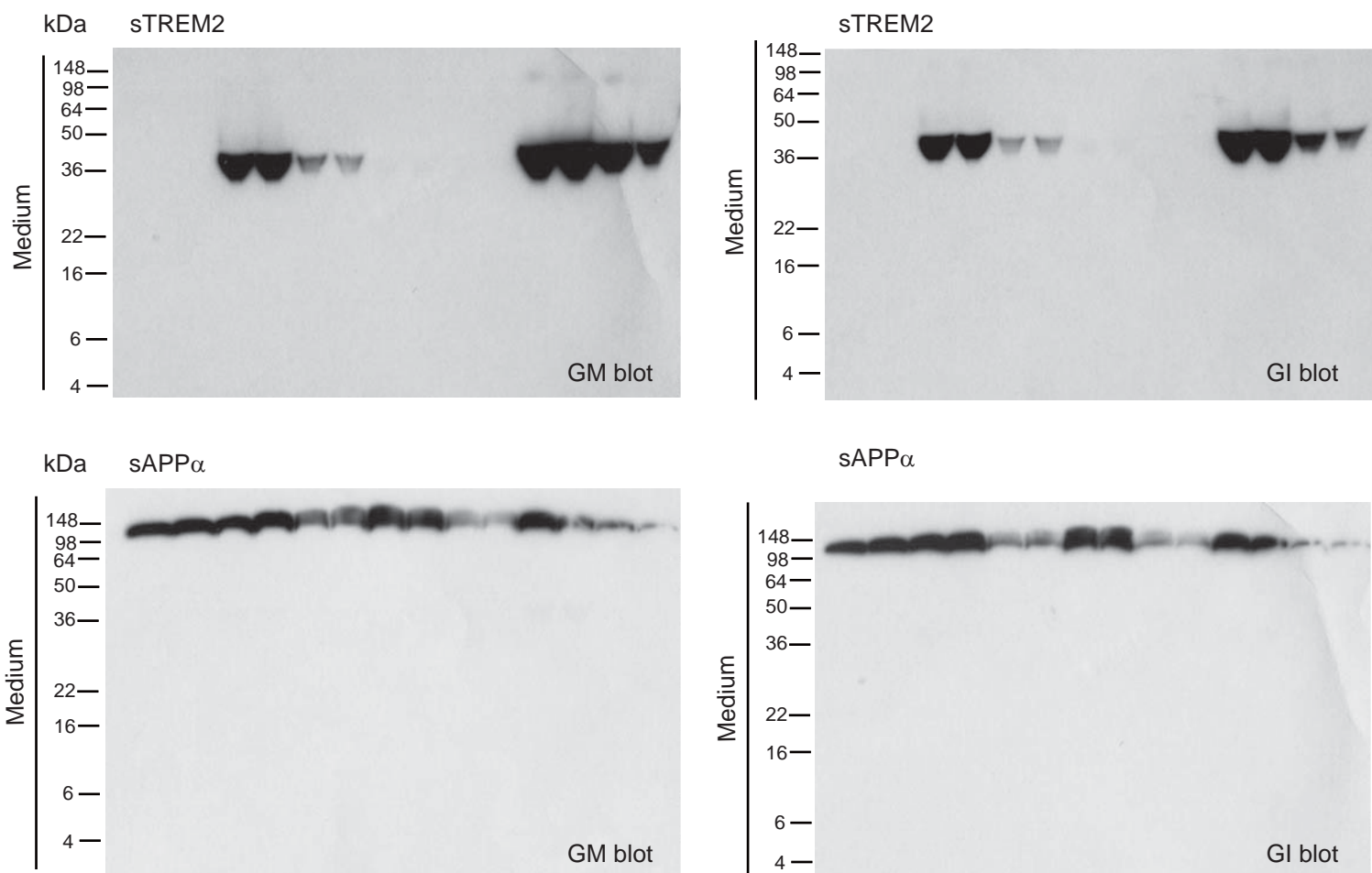**G**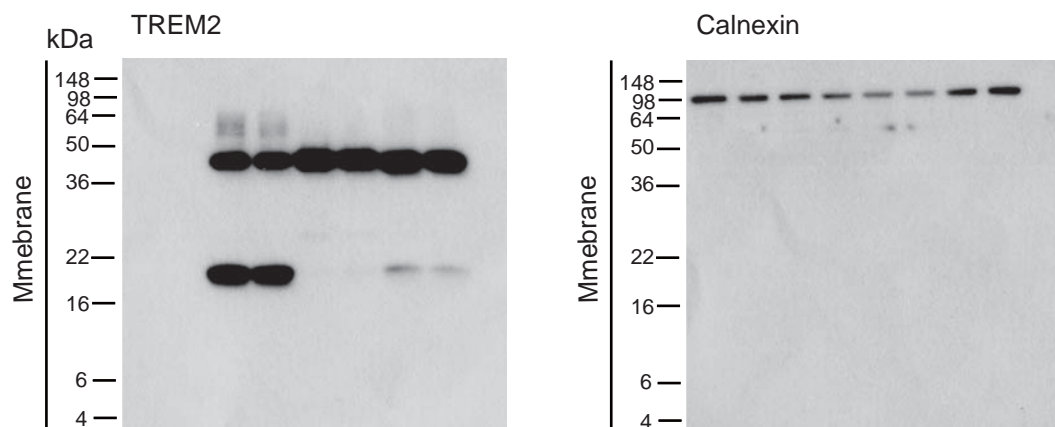

H

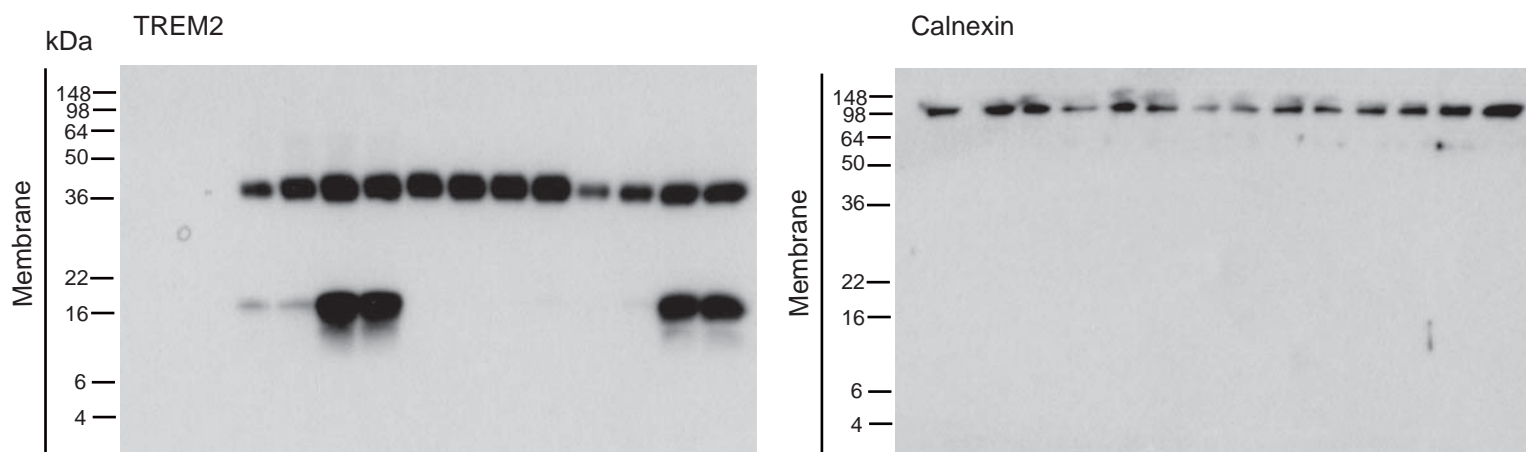

I

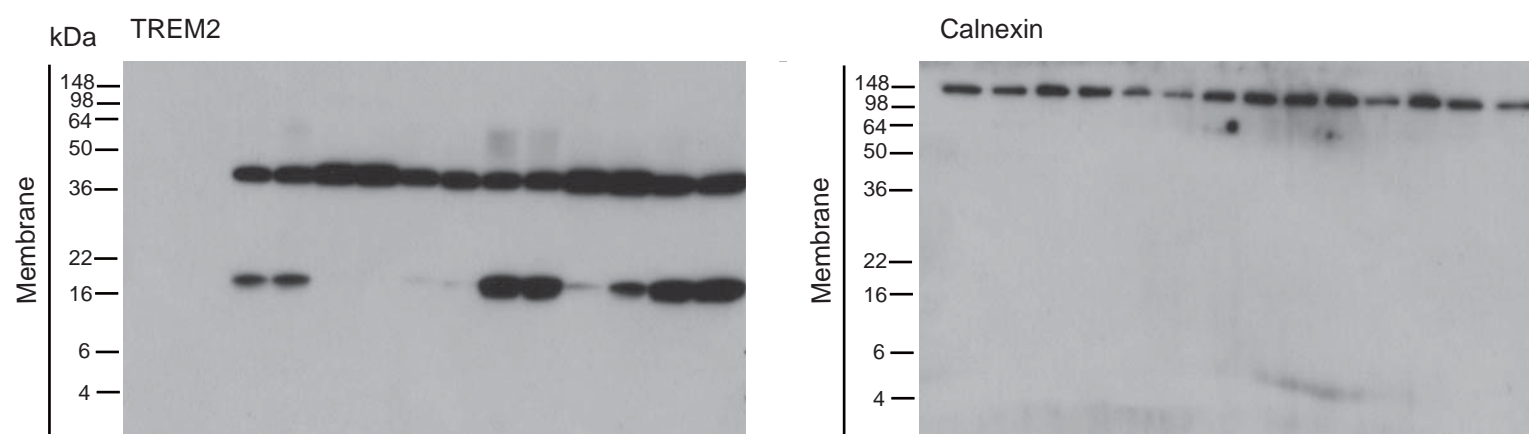

J

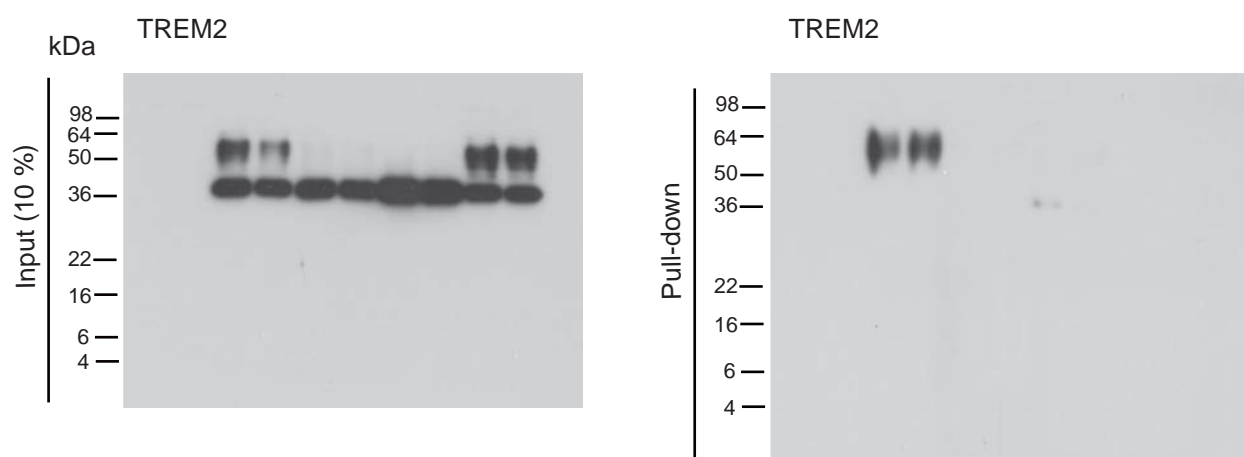

Supplement: Supplementary file 3 — Source Data for Figure 2 [file EMMM-9-1356-s002.pdf]
